# Use of Artificial Neural Networks as a Predictive Tool of Dissolved Oxygen Present in Surface Water Discharged in the Coastal Lagoon of the Mar Menor (Murcia, Spain)

**DOI:** 10.3390/ijerph19084531

**Published:** 2022-04-09

**Authors:** Eva M. García del Toro, Luis Francisco Mateo, Sara García-Salgado, M. Isabel Más-López, Maria Ángeles Quijano

**Affiliations:** 1 Departamento de Ingeniería Civil: Hidráulica y Ordenación del Territorio ETSI Civil, Universidad Politécnica de Madrid Alfonso XII, 3, 28014 Madrid, Spain; sara.garcia@upm.es (S.G.-S.); marian.quijano@upm.es (M.Á.Q.); 2 Departamento de Ingeniería Civil: Construcción, Infraestructura y Transporte ETSI Civil, Universidad Politécnica de Madrid Alfonso XII, 3, 28014 Madrid, Spain; luis.f.mateo@upm.es (L.F.M.); mariaisabel.mas@upm.es (M.I.M.-L.)

**Keywords:** intensive agriculture, eutrophication, nitrates, dissolved oxygen (DO), multiple linear regression (MLR), back-propagation neural network (RPROP)

## Abstract

The Mar Menor is a Mediterranean coastal saltwater lagoon (Murcia, Spain) that represents a unique ecosystem of vital importance for the area, from both an economic and ecological point of view. During the last decades, the intense agricultural activity has caused episodes of eutrophication due to the contribution of inorganic nutrients, especially nitrates. For this reason, it is important to control the quality of the water discharged into the Mar Menor lagoon, which can be performed through the measurement of dissolved oxygen (DO). Therefore, this article aimed to predict the DO in the water discharged into this lagoon through the El Albujón watercourse, for which two theoretical models consisting of a multiple linear regression (MLR) and a back-propagation neural network (RPROP) were developed. Data of temperature, pH, nitrates, chlorides, sulphates, electrical conductivity, phosphates and DO at the mouth of this watercourse, between January 2014 and January 2021, were used. A preliminary statistical study was performed to discard the variables with the lowest influence on DO. Finally, both theoretical models were compared by means of the coefficient of determination (R^2^), the root mean square errors (RMSE) and the mean absolute error (MAE), concluding that the neural network made a more accurate prediction of DO.

## 1. Introduction

Coastal lagoons result from the mixing of fresh and salt water [1]. They represent dynamic and heterogeneous systems, which are subject to intermittent or permanent seawater flows that are integrated through sand barriers while fresh water is incorporated in the form of runoff and discharges from streams or watercourses. The ionic composition of the water in these lagoons is also influenced by that of the groundwater.

In recent times, the strong anthropic activity developed in coastal areas has altered the quantity and nature of these incoming water flows, increasing their irregularity and trophic load [1,2]. Since the 1970s, an increase in the amount of nutrients from agricultural fertilizers has been observed, leading to the eutrophication of many coastal areas of the world [3,4]. Eutrophication is causing major ecological disasters affecting both flora and fauna of the ecosystems associated with coastal lagoons [5,6]. The high amount of biomass produced decreases the availability of light, favoring among the primary producers the most competitive community for light, i.e., phytoplankton, at the expense of macrophytes [7,8]. This overproduction leads to a loss of diversity [9], habitat destruction and mortality by anoxia of various species [10,11,12,13]. In coastal areas, which are characterized by strong population growth, eutrophication has become a serious threat since the 1950s [14]. So, eutrophication is one of the greatest ecological risks to ecosystem health, and one of the greatest challenges to sustainable water management. It is increasingly recognized that eutrophication has multidimensional consequences for water quality, as well as the ecosystem and human health. It even conditions the development of certain economic activities. These consequences depend on site-specific conditions, in particular the ecological stability of the system, types of land use, climate change and the presence of other pollutants [15].

Coastal lagoons are particularly sensitive to eutrophication, as these systems tend to concentrate anthropogenic nutrient inputs due to restricted exchanges with the sea and long water residence time [7]. The Mar Menor area is subject to a great economic activity, which includes a high urban pressure. In the 1970s, the Tajo-Segura water transfer took place, which led to the development of intensive agriculture in the region of Campo de Cartagena, whose main collector towards the lagoon is the El Albujón watercourse. This has caused episodes of eutrophication in the lagoon, characterized by increased turbidity [16] and decreased dissolved oxygen (DO) [17,18].

DO is a parameter that has been a frequently used to assess water quality [19,20,21,22]. Water quality monitoring is very useful to control the health of ecosystems, especially in critical areas where possible pollution episodes are foreseen and/or relevant socioeconomic activities. Surface water quality prediction is a basic task in water resources management studies, to establish the reasons for water quality deterioration and to keep pollution within permissible limits [23,24].

The prediction of different parameters affecting water quality can be performed through multiple linear regression (MLR) models [23,24], or by applying artificial intelligence, which is an effective tool for dealing with the problems of dynamic complex hydrological systems [25,26]. Within artificial intelligence, artificial neural networks (ANN) stand out as very useful models for data processing [27]. An artificial neural network is a computational model whose architecture attempts to mimic the behavioral relationships of the brain. It consists of a limited number of interconnected elements (neurons) distributed in an input layer, one or more hidden layers and an output layer. The input layer has the function of receiving information from the outside, while the neurons of the output layer are in charge of delivering the results of the predictions made by the neural network. Hidden layers generate the chaining relationships between inputs and outputs, extract and refine the relationships and characteristics of the input variables to predict the outputs that are of interest to the study. These types of networks are able to forecast water quality parameters through the relationships between inputs and outputs, without taking into account the internal mechanisms of the forecasting models [28]. In this regard, Ay and Kisi [22] applied three different models to predict the concentration of DO in river water in Foundation Creek, El Paso, Colorado, consisting of two ANN models (MLP (Multilayer Perceptron) and RBNN (Radial Basis Neural)) and one statistical model (MLR). These authors concluded that ANN models fit very well in the estimation of DO, obtaining a much higher accuracy with them than with MLR. On the other hand, Zhang et al. [29] used back propagation neural networks (BPNN) to predict DO as a criterion for water quality assessment, using data of temperature, nitrogen content of the ammonia (NH3-N) and biological oxygen demand (BOD) to simulate DO concentration with an average relative error lower than 8%, obtaining a good predictive tool. Other authors such as Liu et al. [30] used an Elman neural network (ENN) model to predict DO for rapid assessment of Singapore coastal waters. In this study, the designed network architecture consisted of seven or eight hidden layer nodes, and good results for DO were obtained. Wang et al. [31] studied and compared four types of models to predict monthly ammonia nitrogen (NH(4+)-N) and DO in the Harbin region, northeast China. These four models were based on: bootstrapped wavelet neural network (BWNN), ANN, wavelet neural networks (WNN) and AutoRegressive Integrated Moving Average (BANN and ARIMA). The results showed that the BWNN model could handle water quality time series data, which are highly fluctuating and non-seasonal, and produced a better performance than the other four models. Naha et al. [32] investigated the ability of adaptive neurofuzzy inference system (ANFIS) in the Johor river basin, Malaysia, to predict DO concentrations. The results obtained by the ANFIS model were compared to those obtained by the model developed with the technique of multilayer perceptron neural network (MLP-NN), and a higher accuracy for the ANFIS model, both in the prediction of average DO and in its extreme values, was observed. Another parameter that can be used to evaluate the degree of eutrophication in waters is the amount of chlorophyll. In this regard, Jimeno-Saez et al. [33] applied machine learning algorithms to estimate the chlorophyll in seawater from the coastal lagoon of Mar Menor. The algorithms used were Support Vector Regressions (SVRs) and Multilayer Neural Networks (MLNNs), obtaining better results in the validation phase for the SVRs, as well as satisfactory final results for the prediction of chlorophyll concentration.

The purpose of this study is to determine the extent to which the DO concentration in freshwater present in the mouth of the El Albujón watercourse to Mar Menor lagoon, can be forecasted using theoretical models. To do so, a model based on MLR and a model based on ANN were developed. The architecture of the ANN model was developed upon a back-propagation algorithm and using the Knime application, which is an open-source tool, easy to use and requires few computer resources. This will presumably represent an improvement with respect to the literature consulted. Experimental water quality data were used, and the choice of input variables for the modelling was based on a statistical correlation analysis of the field data. Predicted DO concentrations obtained by both models were compared with measured values, to identify the best predictive tool.

## 2. Materials and Methods

### 2.1. Location of Study Area

The Mar Menor is a Mediterranean coastal saltwater lagoon, located in the Region of Murcia, southwestern Spain, with a semi-arid climate, which occupies an area of approximately 170 km, a coastline length of 73 km and a maximum depth of 7 m. This lagoon is separated from the Mediterranean Sea by a sandy coastal bar approximately 19 km long, with different widths, with five channels or gullies through which it exchanges water with the sea, and where a summer colony known as the Manga del Mar Menor is located. The Mar Menor is also a place of vital natural importance, since in its surroundings, up to 10 figures of environmental protection, geological and ecosystemic interest converge. The area is integrated within the Natura 200 Network. It has a ZEPA (Zone of Special Interest for Birds), which includes the Protected Landscape called “Open Spaces and Islands of the Mar Menor”. In addition, the Mar Menor is a Wetland of International Importance (WII), according to the Ramsar Convention, being therefore essential to protect the ecosystems that compose it.

**Figure 1:**
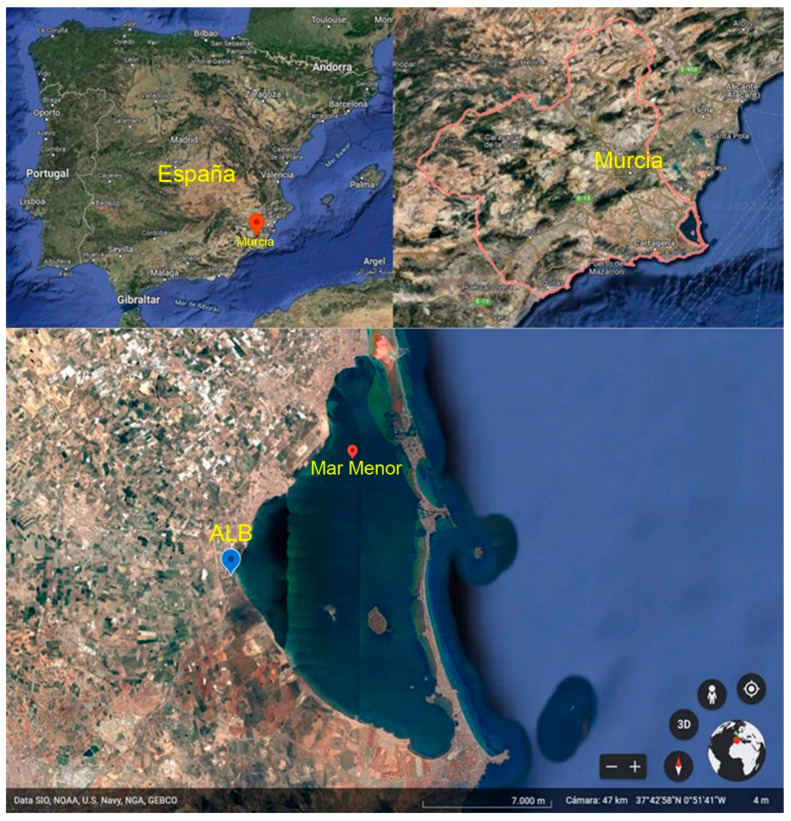
Situation of Region of Murcia and El Albujón watercourse. Adapted with permission from Ref [34]. Copyright 2022. Inst. Geograf. Nacional de España.

The physicochemical parameters of the water from the El Albujón watercourse at its mouth in the Mar Menor lagoon (Figure 1) selected for this study were temperature, pH, nitrates, chlorides, sulphates, electrical conductivity, phosphates and DO, corresponding to the period from January 2014 to January 2021. Water analysis was carried out by the Water Quality Laboratory of the Confederación Hidrográfica del Segura. Temperature, pH, electrical conductivity and DO were determined in situ by electrometric probes following internal methods based on the corresponding Standard Methods (SM2550-B, SM 4500 H, SM 2510-B and SM 4500-O-G, respectively). On the other hand, ex situ parameters such as nitrates, chlorides, sulphates and phosphates, were determined by Ion Chromatography by an internal method based on UNE EN-ISO 14911.

### 2.2. Development of Mathematical Models

#### 2.2.1. Preliminary Statistical Study to Analyze the Influence of Different Variables on DO Concentration

First, the influence of the different variables on DO concentration was analyzed in order to select the variables with the greatest influence and discard those with the least influence. For this purpose, the Pearson coefficients of each parameter were analyzed with DO concentration, using the totality of the data in the period studied.

#### 2.2.2. Multiple Linear Regression (MLR) Model

Once the most influential variables (independent variables) were selected, the MLR method was applied to DO (dependent variable). The goodness-of-fit of the model was verified through the assumptions of linearity, independence, homoscedasticity and normality of the residuals. The hypotheses of the model were tested to see if they fit the data collected.

#### 2.2.3. Artificial Neural Network (ANN) Model

In order to develop the architecture of the ANN-based DO prediction model, different configurations of number of neurons and hidden layers were studied. The starting point was the total of the 153 measurements or data of the variables with the greatest influence according to the preliminary statistical analysis. The ANN was created in two phases, which were the training phase and the model validation phase. The objective was to verify that the proposed network architecture is capable of adequately modelling the simulation target. Normally the network weights are initialized as random values.

During the training or learning phase, the dataset containing both the desired input and output information were processed to optimize the network outputs in order to minimize the error between the objective values and the model outputs. In this study, the data set was divided into two different subsets, so that a first subset of data was used to create a model to serve as a training set for the network. The second subset was used to validate the model created in the previous phase (test set).

The learning algorithm used was the back-propagation algorithm (RPROP). This type of neural network uses a back-propagation procedure, which is a method of learning from a predefined set of inputs and outputs, using a propagation cycle. The ANN architectures for predicting the DO were obtained using the Knime application, a tool selected because it is open source and easy to use and parameterize. In addition, to avoid scale differences between the physical variables involved in the network, a normalization process was carried out. In this process, each variable was transformed to a natural system between 0 and 1.

To find the best data distribution ratio for the training test and the model validation test, respectively, the network was experimented and run with different data distribution percentages. Thus, the training/test ratios tested were 90–10% to 50–50%, decreasing and increasing, respectively, by 5% for each test.

Different combinations of hidden layers and number of neurons per layer were also tested during the network development process. Processes were tested with 5, 4, 3, 2 and 1 hidden layers and combinations from 20 to 100 neurons per layer, respectively. Between all the combinations tested, the one considered most efficient was chosen, consisting of 3 hidden layers and 50 neurons per layer.

**Figure 2:**
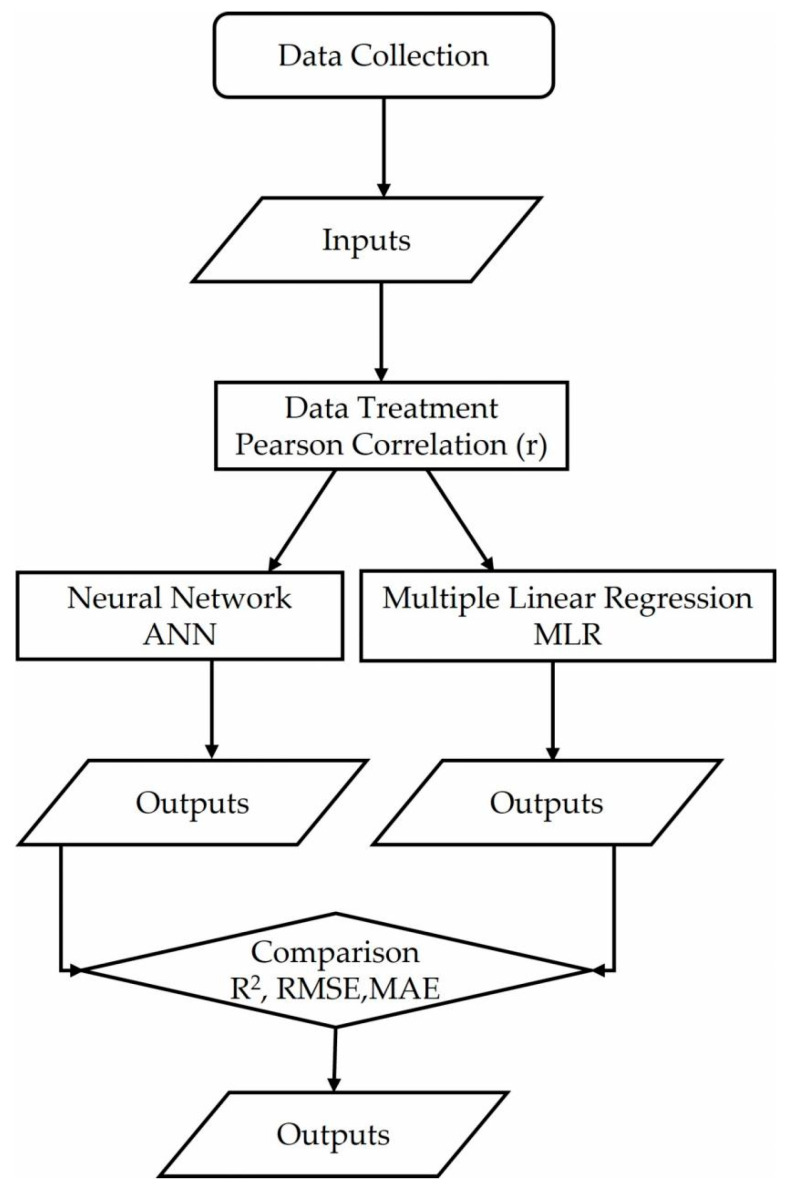
General process flow chart.

**Figure 3:**
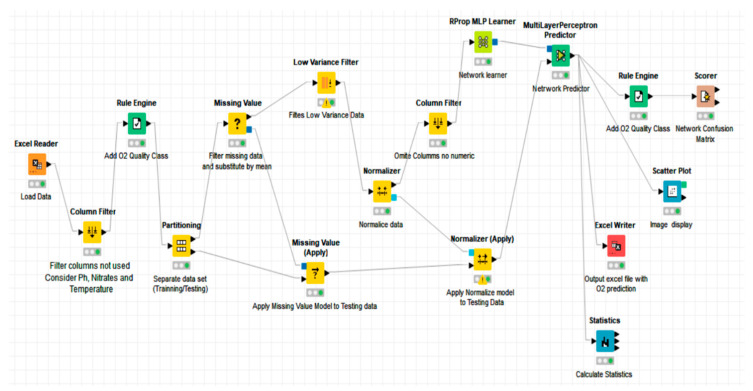
ANN flow chart.

As a summary, two flow charts are included. Figure 2 corresponds to the general process and Figure 3 represents the neural network’s flow chart.

## 3. Results and Discussion

Table 1 shows the physicochemical variables used in the present study, together with the minimum, maximum, arithmetic mean and standard deviation values of the data provided by the Consejería de Agua, Agricultura, Ganadería, Pesca y Medio Ambiente of Region of Murcia and the Confederación Hidrográfica of Segura river.

### 3.1. Preliminary Statistical Study to Analyse the Influence of Different Variables on DO Concentration

The selection of the variables with the greatest influence on DO concentration in the freshwater of the El Albujón watercourse was carried out through Pearson correlation studies. Table 2 shows the results obtained.

From the Pearson correlation coefficients obtained, it was concluded that temperature and pH presented a significant correlation on DO concentration in the analyzed waters (*p* < 0.05). Likewise, nitrate concentration was also included in the models developed, because compared to the other variables, it presented a difference approximately 10 times greater than the rest of the variables. In addition, it is widely known that eutrophication episodes, which cause a decrease in the DO in a given body of water, are due to the presence of high concentrations of nutrients, such as nitrates, so in this study it is considered as a relevant variable. The rest of the variables (chlorides, sulphates and electrical conductivity) can be discarded from the model, because they did not have a significant influence based on the low values obtained for their respective Pearson correlation coefficients (r), which is related to the characteristics of the water studied, which is a freshwater.

### 3.2. MLR Model

From the variables selected in the preliminary statistical study, the MLR model was developed with DO as the independent variable, and nitrate concentration, temperature and pH as dependent variables. The summary of the MLR model is shown in Table 3.

The results obtained indicated that DO is explained in an extension of 44% by the variables temperature, pH and nitrate concentration, according to the MLR model considered.

The equation that fits the data were as follows:

(1) DO=0.157+0.6313 pH−0.281 T+0.27 N

where

DO: Dissolved oxygen,

pH: pH value,

T: Temperature,

N: Nitrate concentration,

To verify that these variables can be related using the proposed MLR model, the verification of the assumptions of linearity was checked, together with the independence, homoscedasticity and normality of residuals.

#### 3.2.1. Linearity

Table 4 shows the results of the ANOVA analysis of the MLR model, and it can be concluded that the linearity assumption is met because the *p*-value is significant because it is lower than the significance level (α < 0.05).

#### 3.2.2. Independence

According to the value obtained for the test of independence of the Durbin–Watson residuals, shown in Table 3, these can be considered independent because this value is within the range 1.5–2.5.

#### 3.2.3. Homoscedasticity

To test the model homoscedasticity, the correlation between the absolute value of the residuals and their estimated values was calculated. The results are shown in Table 5.

The results obtained showed that the *p*-value obtained was higher than the level of significance (α = 0.05), so the null hypothesis H0 was accepted, and it can be affirmed that there is no correlation between the variables. Therefore, the model homoscedasticity was proved.

#### 3.2.4. Normality

To check the assumption of normality of the residuals, the Kolmogorov–Smirnov (K-S) test was performed. The results of this test are shown in Table 6.

As it can be observed, the *p*-value is 0.200, so it is higher than 0.05 and it is assumed that the variable is distributed according to a normal probability function.

Therefore, from the results obtained regarding the linearity and characteristics of the residuals, it can be concluded that the MLR model can be a valid prediction model for DO as a function of pH, temperature and nitrate concentration.

### 3.3. ANN Model

**Figure 4:**
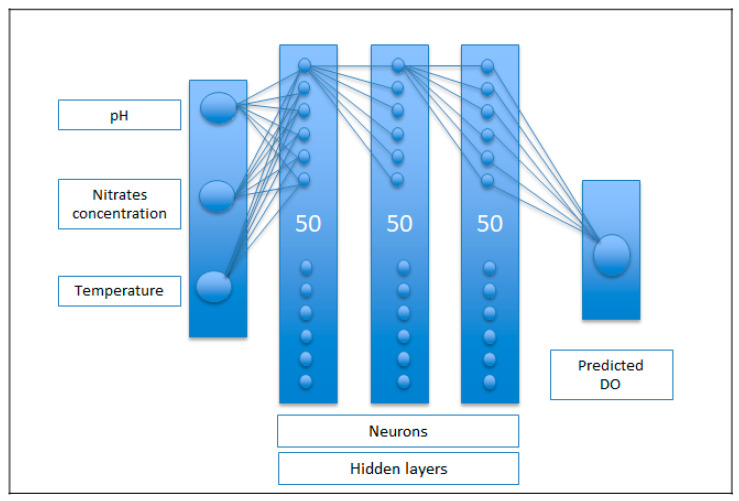
ANN architecture proposed for the prediction model of the DO present in the waters of the mouth of the El Albujón watercourse.

The ANN architecture proposed for the prediction model of the DO present in the waters of the mouth of the El Albujón watercourse in the Mar Menor is shown in Figure 4.

From the study of the distribution ratio of the data for training and validation of the model, it was found that the optimum distribution to obtain the best results with the model was as follows: 70% training data and 30% validation data. This data distribution was also the most efficient, since it minimizes execution times and the prediction showed the lowest error.

### 3.4. Comparison between the MLR and ANN Models

The comparison between the theoretical models developed was based on the calculation of three statistical parameters: the mean square error (RMSE), the mean absolute error (MAE), and the coefficient of determination R^2^. The best method will be by presenting the minimum values of RMSE and MAE, and the R^2^ value closer to 1.

**Figure 5:**
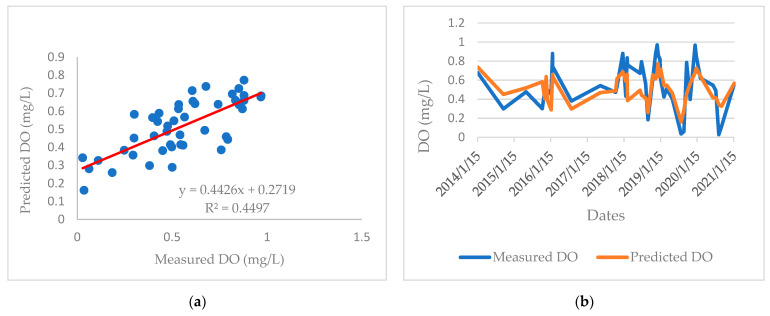
Comparison between experimental DO data and those obtained by the proposed MLR model. (**a**) Correlation between measured and predicted DO; (**b**) Date profile of measured and predicted DO.

**Figure 6:**
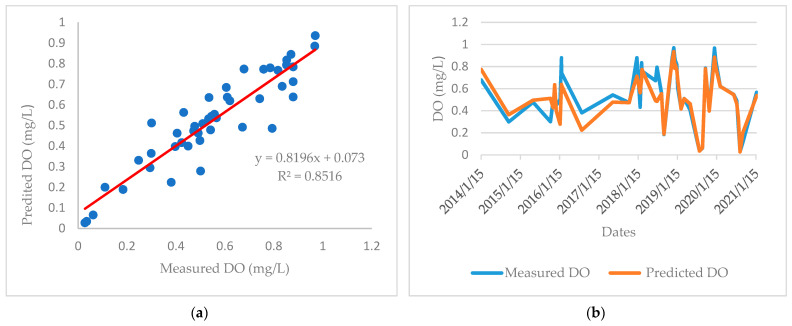
Comparison between experimental DO data and those obtained by neural network method (**a**) Correlation between measured and predicted DO; (**b**) Date profile of measured and predicted DO.

Figure 5 and Figure 6 show the experimental data of DO present in the waters of the mouth of the El Albujón watercourse, and the data that can be predicted by the proposed MRL model and ANN model, respectively.

Table 7 shows the RMSE, MAE and R^2^ values of the proposed models.

As it can be observed, the ANN model presented a coefficient of determination value closer to 1 than the MRL model (0.85 versus 0.44), as well as the lowest values of RMSE and MAE. Therefore, it can be concluded that a higher estimation accuracy was achieved by the ANN model than by the MLR model. This can also be observed in the curve profiles shown in Figure 2B (MLR model) and Figure 3B (ANN model).

Other authors have also concluded that neural networks are effective methods for the theoretical determination of DO in both lakes and rivers. This is the case of, for example, Ay and Kisi [22], who designed a neural network using four variables, such as pH, temperature, electrical conductivity and flow rate, obtaining satisfactory results for the determination of DO in a river in El Paso, Colorado. In the present work, the preliminary statistical study showed that electrical conductivity was not relevant in the variation of DO in the water discharged into the Mar Menor lagoon, through the El Albujón watercourse. On the other hand, it can be highlighted that the main contribution of this paper is the method used to create the ANN model, since the neural network architectures for predicting DO were obtained using the Knime application, which is an open-source tool, selected because it is easy to use and requires few computer resources.

## 4. Conclusions

The DO concentration in the waters can be estimated by theoretical models as a function of different physicochemical variables. In this study, available data regarding water quality were: temperature, pH, nitrates, chlorides, sulphates and electrical conductivity. The preliminary statistical analysis carried out showed that the variables that most affect the variation of DO in the studied waters were temperature, pH and nitrate concentration.

Based on these three variables, two theoretical models were developed to estimate the DO in the waters of the mouth of the Albujón watercourse, using an MRL model and a back-propagation ANN model, which is a method of learning from a predefined set of inputs and outputs, using a propagation cycle, which finally led to an improvement of the model. On the other hand, the Knime application was used to develop the neural network architectures. This application is an open-source tool, easy to use and requires few computer resources. However, it presents the drawback that a lower precision can be obtained in the predicted values, but in the present work, a commitment situation between computer resources, time and precision was reached, and satisfactory results were obtained.

Between both methods, the one that provided a higher precision in the DO results was the ANN model, which presented a coefficient of determination, R^2^, of 85.16%, compared to the 44.43% obtained by the MLR model. Regarding the errors, both the mean square error and the mean absolute error were lower in the case of the ANN model.

Therefore, it can be affirmed that the neural network designed, using the back-propagation model and the Knime application, was a satisfactory method to predict the variation of DO in the waters studied, and can be a useful, economical and effective tool to collaborate in the management of the water quality in an area as sensitive as the Mar Menor coastal lagoon in Murcia, Spain.

## Figures and Tables

**Table 1:** Physicochemical variables available to develop the theoretical models, at the mouth of El Albujón watercourse, between January 2014 and January 2021.

Parameter	Unit	Min	Max	Mean	Standard Deviation	*n*
Temperature	°C	8.8	27.8	19	5	153
pH		6.8	8.7	8.0	0.3	153
Nitrates	mg/L	1.3	311	15 × 10^1^	5 × 10^1^	153
Chlorides	mg/L	25.8	5146	23 × 10^2^	9 × 10^2^	153
Sulphates	mg/L	41	4808	23 × 10^2^	7 × 10^2^	153
Electrical conductivity	μS/cm	2098	18410	9 × 10^3^	3 × 10^3^	153
Dissolved oxygen (DO)	mg/L	2.21	15.0	9	8	153

**Table 2:** Values obtained for the Pearson correlation coefficients between DO and different variables.

Pearson Correlation (r)	
Variables	DO
Chlorides	−0.067
Nitrates	0.188
Sulphates	−0.038
Temperature	−0.507
pH	0.540
Electrical conductivity	0.017

**Table 3:** Summary of the MLR model developed.

R	R^2^	Adjusted R^2^	Standard Error	Durbin-Watson
0.66	0.44	0.43	0.1625024	1.541

**Table 4:** Results of the ANOVA analysis of the MLR model.

Model	Sum of Squares	df	Mean Square	F	Sig.
Regression	3.45	3	1.048	39.703	0.000
Residuals	3.94	149	0.26		
Total	7.08	142			

**Table 5:** Correlation between the absolute value of the residuals and their estimated values.

		ABS Residuals	Predicted Value
ABS Residuals	Pearson correlation coefficient	1	0.054
	*p*-value		0.509
	N	153	153
Predicted Value	Pearson correlation coefficient	0.054	1
	*p*-value	0.505	
	N	153	153

**Table 6:** K-S Test Results.

		Unstandardized Residual
N		153
Normal parameters	Mean	0.00000000
	Deviation	0.16089077
Most extreme differences	Absolute	0.043
	Positive	0.043
	Negative	−0.035
Kolmogorov–Smirnov Z		0.043
*p*-value		0.200

**Table 7:** RMSE, MAE and R^2^ values of the MRL model and the ANN model.

Model	RMSE	MAE	R^2^
MLR	0.159842825	0.130820391	0.4443
ANN	0.140726705	0.102803977	0.8516
